# Nitrogen-doped, FeNi alloy nanoparticle-decorated graphene as an efficient and stable electrode for electrochemical supercapacitors in acid medium

**DOI:** 10.1186/s11671-015-0778-6

**Published:** 2015-03-01

**Authors:** Ahmed G El-Deen, Mohamed El-Newehy, Cheol Sang Kim, Nasser AM Barakat

**Affiliations:** Bionanosystem Engineering Department, Chonbuk National University, Jeonju, 561-756 Republic of Korea; Petrochemical Research Chair, Department of Chemistry, College of Science, King Saud University, Riyadh, 11451 Saudi Arabia; Department of Chemistry, Faculty of Science, Tanta University, Tanta, 31527 Egypt; Chemical Engineering Department, Faculty of Engineering, El-Minia University, El-Minia, Egypt; Organic Materials and Fiber Engineering Department, Chonbuk National University, Jeonju, 561-756 Republic of Korea

**Keywords:** FeNi alloy, Graphene, Electrochemical supercapacitors, Specific capacitance

## Abstract

Nitrogen-doped graphene decorated by iron-nickel alloy is introduced as a promising electrode material for supercapacitors. Compared to pristine and Ni-decorated graphene, in acid media, the introduced electrode revealed excellent specific capacitance as the corresponding specific capacitance was multiplied around ten times with capacity retention maintained at 94.9% for 1,000 cycles. Briefly, iron acetate, nickel acetate, urea, and graphene oxide were ultrasonicated and subjected to MW heating and then sintered with melanin in Ar. The introduced N-doped FeNi@Gr exhibits remarkable electrochemical behavior with long-term stability.

## Background

Due to the intense shortage of reserves of fossil fuels, increasing the awareness of energy conservation and environmental preservation has internationally spurred unprecedented interest in developing green and renewable energy. Electrochemical capacitors (ECs), as one of the promising technology for energy storage systems, have attracted significant attention due to their high power performance, very long life cycle, and reasonable energy density. Accordingly, ECs are often favored in various applications including portable electronics, uninterruptible power sources, memory backup systems, and hybrid electric vehicles [1-3]. It is accepted that the formation of electric double layer depends mainly on the physicochemical properties of the electrode material [4].

As for other energy devices such as fuel cells and dye-sensitized solar cells, the best performance in the electrochemical supercapacitors was achieved with precious metal-based electrodes which constraints the commercialization process [5,6]. Recently, some bimetallic transition metal nanostructures showed good performances as electrodes in the energy devices [7-12]; however, according to our best knowledge, this promised structure has been rarely exploited as a supercapacitor [4]. Recently, carbonaceous materials have been widely utilized as support for functional materials, and distinct enhancement in the specific capacitance was observed [13,14]. Graphene is a fascinating carbon material having marvelous characteristics, so it was incorporated with the most promising electrode materials for electrochemical energy storage [15,16].

It is generally believed that pseudocapacitance in carbon-based materials is largely based on the redox reactions of surface quinoid functionalities, whose reduction requires protons to proceed [17]. Nevertheless, some other oxygenated functional groups might be electrochemically active at the working potentials of the acidic solutions, such as some pyrone groups [18]. Therefore, and as may be expected, faradaic phenomena have a large dependence on the pH of the solution. In this sense, enormous differences in capacitance values depending on the electrolyte used can be found. As a general trend, for the same active electrode material, higher capacitance values can be observed in the acidic media compared to those obtained in the basic media [19,20]. On the other hand, nitrogen-doped carbon nanostructural supports exhibited significantly high electrochemical properties and distinct stability because of owning high surface nucleation sites which allow the anchorage and high dispersion of the catalyst nanoparticles on the support surface material [21-23]. Moreover, nitrogen doping improves the durability of the resultant carbon support catalysts because of the enhanced *π* bonding [24,25] and the basic properties due to the strong electron donor behavior of nitrogen atoms [26]. The work carried out by Pietrzak et al. reported that samples containing significant amounts of nitrogen showed higher capacitance values due to the presence of pseudofaradaic reactions of the nitrogen functional groups [27].

Besides, some transition metal-based materials containing manganese, silver, nickel, and iron revealed good electrochemical behavior [28-32]. In this study, the proposed N-doped FeNi@Gr structure strongly enhanced the specific capacitance, stability, and recyclability compared to pristine and Ni-decorated graphene.

## Methods

### Procedure

Nitrogen-doped, FeNi alloy-doped graphene was synthesized by dissolving 250 mg of urea in 100 ml aqueous solution from iron(II) acetate (0.5 mM) and nickel(II) acetate tetrahydrate (0.5 mM); the solution was stirred for 2 h and ultrasonicated for 30 min. The obtained solution was mixed with 50 ml aqueous solution containing 200 mg of graphene oxide (GO) prepared by a modified Hummers method [33] using microwave for 2 min at 600 W for thermal exfoliation. After mixing the two solutions, the obtained slurry refluxed for 12 h at 150°C. After filtration, the obtained solid material was grinded with double amount of melanin and sintered under Ar atmosphere for 4 h at 750°C.

### Characterization

A Rigaku X-ray diffractometer (Rigaku Co., Tokyo, Japan) with Cu Kα (*λ* = 1.54056 Å) radiation over a range of 2*θ* angles from 10° to 80° was utilized to get information about the phase and crystallinity. The surface morphology and elemental mapping were investigated by a JEOL JSM-5900 scanning electron microscope (JEOL Ltd., Akishima-shi, Japan) and transmission electron microscope (TEM, JEOL JEM-2010) operated at 200 kV equipped with energy-dispersive X-ray (EDX) analysis. Electrochemical analysis was carried out using a different three-electrode system: platinum wire as counter electrode, Ag/AgCl as reference electrode, and the fabricated materials as working electrode. The electrochemical impedance spectroscopy (EIS) was measured with a frequency range between 0.01 Hz and 100 kHz. This system was controlled using a VersaStat4 potentiostat device (Princeton Applied Research, Oak Ridge, TN, USA). The surface composition was detected by X-ray photoelectron spectroscopy (XPS) analysis (AXIS Nova, Kratos Analytical Ltd., Manchester, UK) with the following conditions: base pressure 6.5 × 10^−9^ Torr, resolution (pass energy) 20 eV, and scan step 0.05 eV step^−1^.

## Results and discussion

**Figure 1:**
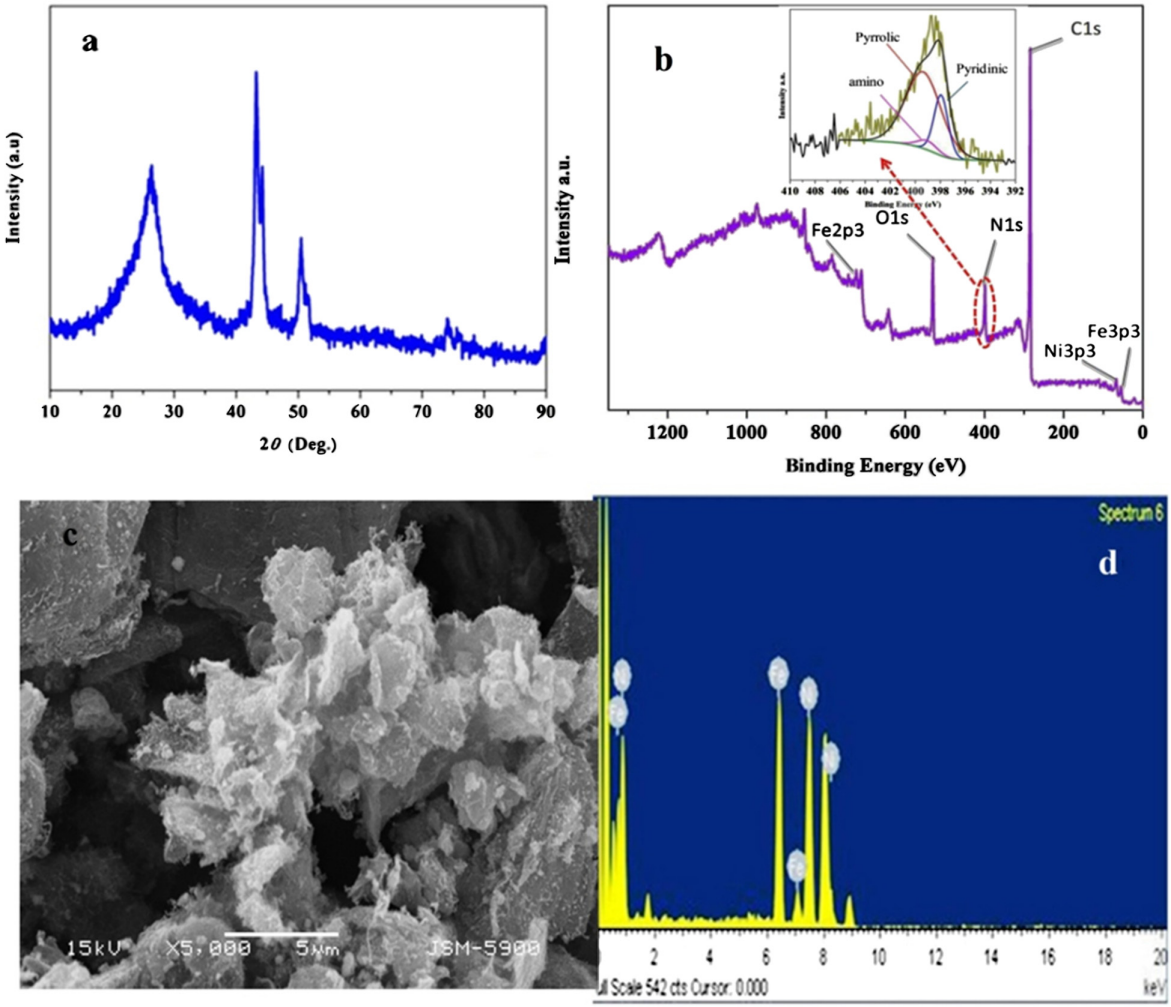
**XRD pattern, XPS spectra, FE-SEM image, and EDX.** XRD pattern **(a)**, XPS spectra **(b)**, FE-SEM image **(c)**, and the corresponding EDX **(d)** for the N-doped FeNi@Gr after the calcination process.

**Figure 2:**
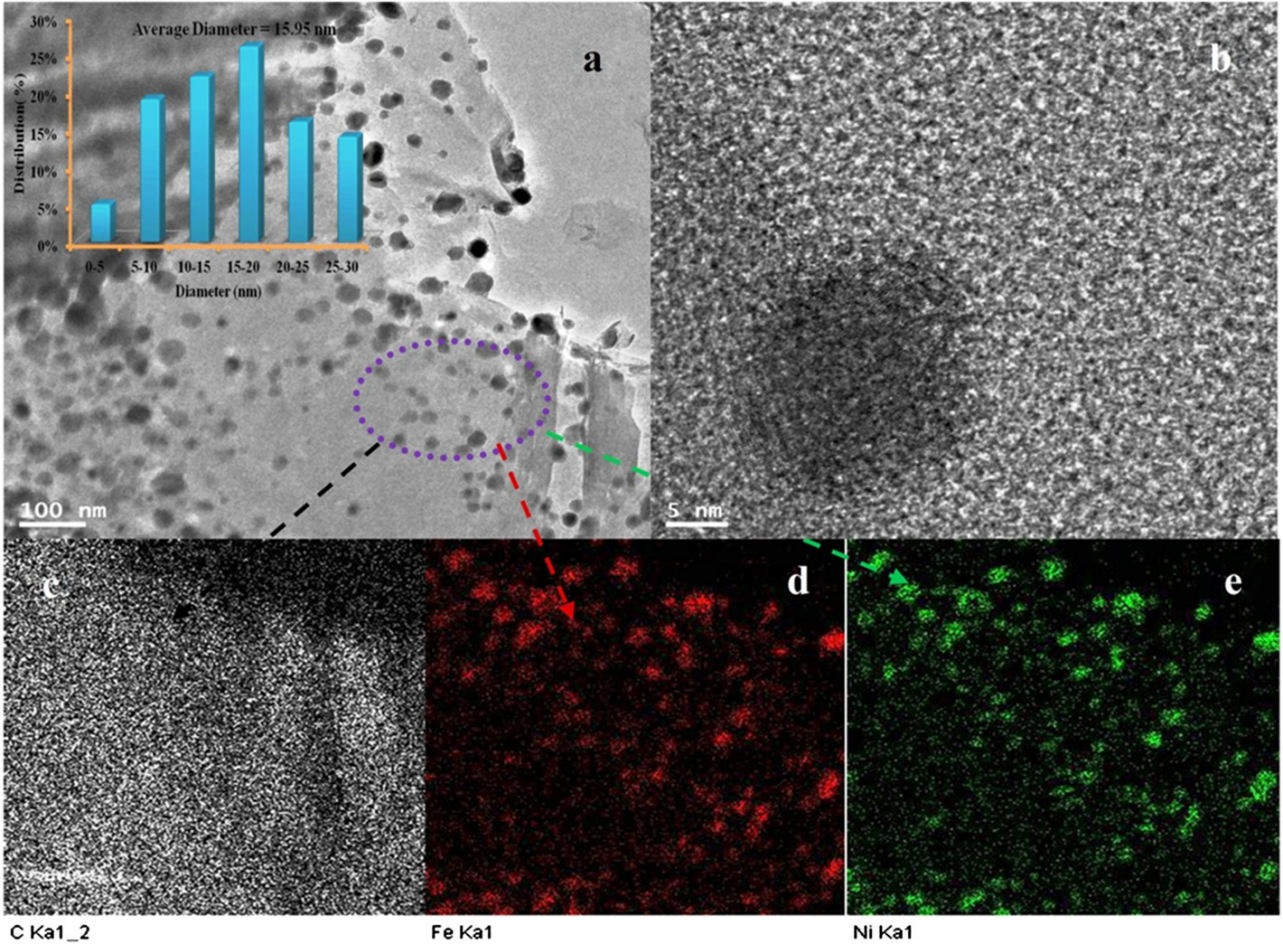
**TEM, HRTEM, and elemental mapping images.** TEM image and average size (inset) of FeNi alloy NPs **(a)**, HRTEM **(b)**, and elemental mapping images (**(c)** carbon, **(d)** iron, and **(e)** nickel) for the marked graphene sheet.

Figure 1a displays the XRD spectra of the anchoring FeNi alloy nanoparticles (NPs) on graphene sheets after the calcination process. The broad diffraction peak observed at 2*θ* = 22.2° to 26.8° indicates the disordered stacking of graphene sheets; however, three distinctive diffraction peaks at 2*θ* values of 43.8°, 51.1°, and 75.6° corresponding to (111), (200), and (220) crystal planes, respectively, indicate the formation of FeNi alloy [34]. XPS spectra presented in Figure 1b confirm the successful doping with nitrogen contents up to 10.1%. Furthermore, the inset showing the high-resolution N1s spectra reveals the presence of nitrogen atoms with three different binding energies, indicating that there are at least three typical nitrogen states: pyridinic (ca. 398 eV), amino (ca. 399.05 eV), and pyrrolic (ca. 399.63 eV) [35]. Figure 1c shows FE-SEM images of the synthesized modified graphene. As shown in the figure, the intercalated FeNi NPs into graphene have a small size and a very good uniform distribution on the graphene sheets. Moreover, the EDX pattern in Figure 1d elucidates the presence of C, Fe, and Ni elements in the investigated area. Figure 2a describes the TEM image of the ultrathin wrinkled graphene few layers. As shown in the inset, the average diameter of the metallic NPs distributed on graphene sheets is approximately 15 nm. The HRTEM image (Figure 2b) indicates that the bimetallic NPs have good crystallinity. Moreover, as shown in the elemental mapping results (Figure 2c,d,e), Fe and Ni have the same distribution that verifies the aforementioned hypothesis about formation of FeNi alloy NPs attached with graphene nanosheets.

**Figure 3:**
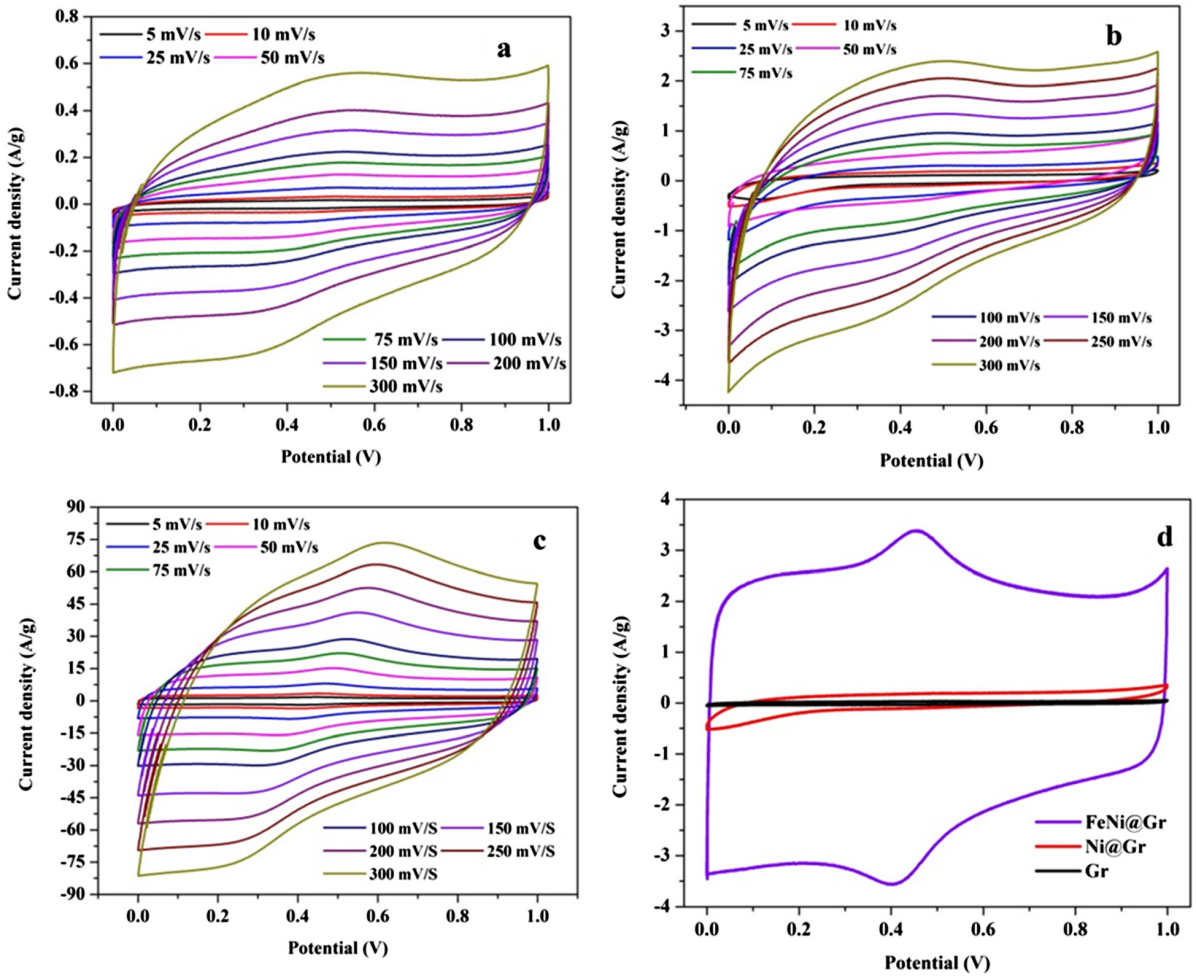
**CV curves.** CV curves of Gr **(a)**, N-doped Ni@Gr **(b)**, and N-doped FeNi@Gr **(c)** in 1 M H2SO4 solution at different scan rates, and comparison CV for the fabricated materials at a sweep rate of 10 mV s^−1^ **(d)**.

**Figure 4:**
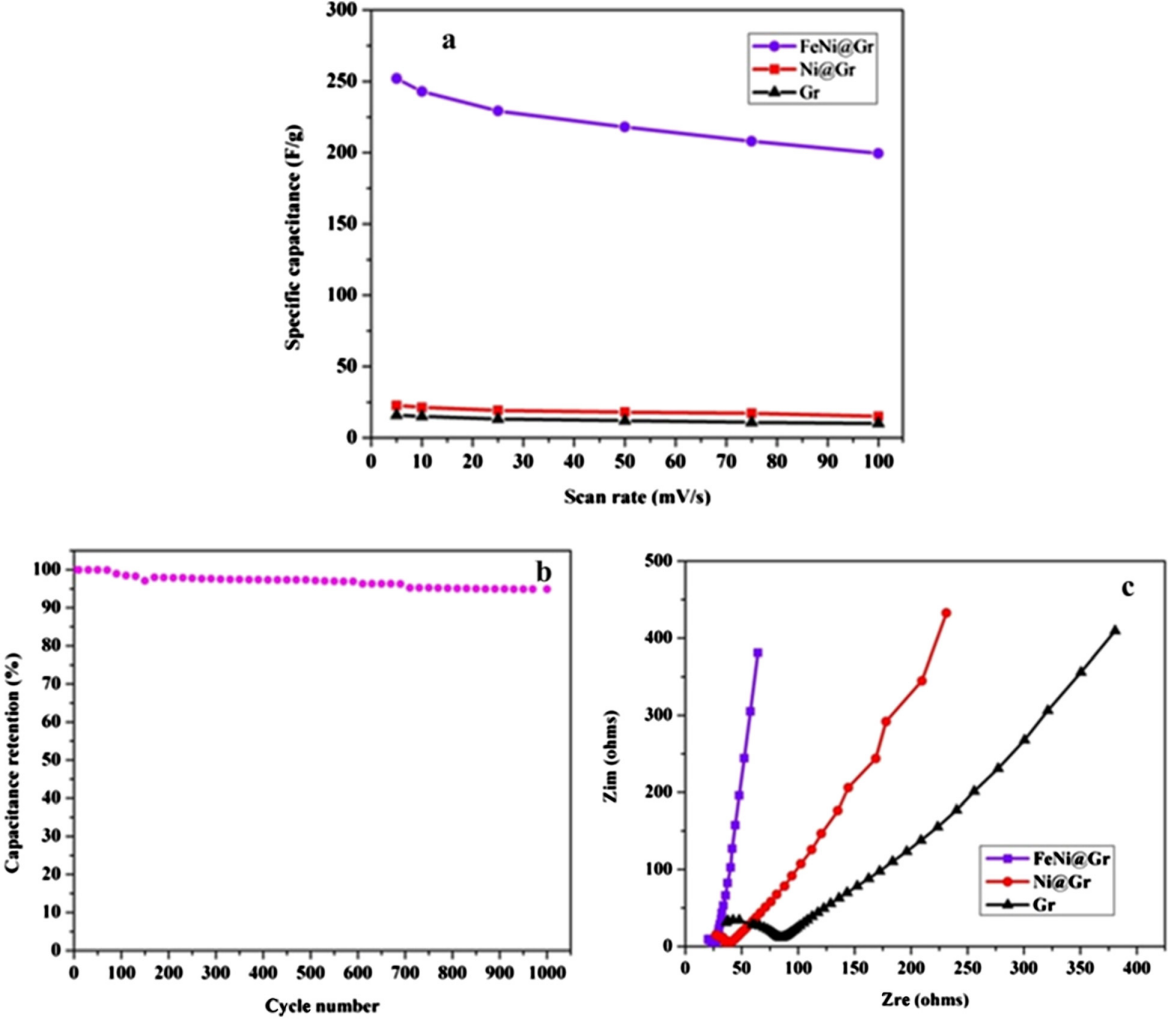
**Specific capacitance, cycling stability, and Nyquist plots.** Specific capacitance for the fabricated materials at different sweep rates **(a)**; cycling stability plots at 50 mV s − 1 **(b)**, and Nyquist plots of N-doped FeNi@Gr, N-doped Ni@Gr, and pristine graphene electrodes in 1 M H2SO4 aqueous solution at different sweep rates **(c)**.

For proper comparison, the electrochemical properties of the introduced N-doped FeNi@Gr electrode, pristine graphene, and N-doped Ni@graphene were investigated. Figure 3 presents typical electrochemical characterization in 1.0 M H_2_SO_4_ as electrolyte solution. As shown in Figure 3a,b,c, all capacitance-voltage (CV) curves exhibit nearly symmetrical rectangular shapes, indicative of an ideal capacitor behavior. The N-doped FeNi@Gr electrode reveals great increase in current density reaching to 100 times that of pristine graphene and 50 times that of N-doped Ni@graphene. Moreover, CV curves retain a relatively rectangular shape without obvious distortion with increasing potential scan rates, even at a scan rate of 300 mV s^−1^. Figure 3d depicts CV profiles for the introduced materials at 10 mV s^−1^. Obviously, the N-doped FeNi@Gr electrode shows redox peaks, indicating that the capacitance characteristics are mainly governed by faradaic redox reactions; thus, the curve pronounces pseudocapacitive behavior attributed to nature-doped materials with nitrogen-containing surface functional groups [36]. Noteworthy, the CV curves become more alike to the rectangular shape, indicating the important role of graphene for the capacitance of the composite. The rate capabilities of the introduced composite were measured at scan rates ranging from 5 to 300 mV s^−1^. The specific capacitance could be determined from the following equation [37,38]:

1 $$ {C}_{\mathrm{sp}}=\frac{{\displaystyle \int }IdV}{2\upsilon m} $$

where *C*_sp_ is the specific capacitance (F g^−1^), *I* is the response current (A), *V* is the potential (V), *υ* is the potential scan rate (V s^−1^), and *m* is the mass of the electroactive materials in the electrodes (g). As shown in Figure 4a, the bimetallic alloy structure achieved exceptional specific capacitance compared to the reported metal- and metal oxide-decorated graphene as the corresponding specific capacitance at 5 mV s^−1^ is 254 F g^−1^ which is about tenfolds of the other formulations. As the introduced material is based on pristine metals and the electrochemical property investigations have been carried out in acidic media, cycling performance is important to check the corrosion resistance of the introduced alloy NPs. A long-term cycle stability test was evaluated for 1,000 cycles (Figure 4b). It can be clearly claimed that the introduced electrode exhibited lossless performance in specific capacitance as after 1,000 cycles (the analysis time was more than 11 h), the specific capacitance maintained at 96.4% from the original value. This finding indicates excellent capacity retention and better long-term cycling stability. EIS confirmed the fast ion transport within the introduced N-doped FeNi@Gr electrode, as shown in Figure 4c. As shown, Nyquist plots demonstrate that the introduced modified graphene at the high-frequency region has the nearest intersecting point on the real axis that represents equivalent series resistance (ESR), indicating that the N-doped FeNi@Gr electrode has low combination resistance of ionic resistance of the electrolyte, intrinsic resistance of the active materials, and small contact resistance between the active material and the current collector compared to other formulations [39]. This difference is attributed to the higher reactivity and faster reaction kinetics and electrode conductivity. The interesting finding is that the intercalating of FeNi alloy into the graphene composite leads to improvement in the conductivity which contributed to pseudocapacitance. Moreover, the smallest semicircle in the high frequency range indicates the FeNi@Gr electrode has a much lower charge transfer resistance and ion diffusion resistance [40,41].

## Conclusions

Bimetallic N-doped FeNi@graphene can be prepared by multi-treatment of FeAc, NiAc, urea, and GO followed by calcination of the mixture with melanin in an argon atmosphere at 750°C. The FeNi alloy structure can distinctly enhance the electrochemical properties compared to the pristine metals. The synthesized alloy NPs have an average size of 15 nm and homogeneous distribution on the graphene sheets. The introduced electrode material provides a high specific capacitance with excellent cyclability in acidic media, so it can be recommended for commercial application.

## References

[1] Winter M, Brodd RJ (2004). What are batteries, fuel cells, and supercapacitors?. Chem Rev.

[2] Simon P, Gogotsi Y (2008). Materials for electrochemical capacitors. Nat Mater.

[3] Miller JR, Simon P (2008). Electrochemical capacitors for energy management. Sci Mag.

[4] Barakat NAM, El-Deen AG, Shin G, Park M, Kim HY (2013). Novel Cd-doped Co/C nanoparticles for electrochemical supercapacitors. Mater Lett.

[5] Xu J, Wang Q, Wang X, Xiang Q, Liang B, Chen D (2013). Flexible asymmetric supercapacitors based upon Co9S8 nanorod//Co3O4@RuO2 nanosheet arrays on carbon cloth. ACS Nano.

[6] Wu ZS, Wang DW, Ren W, Zhao J, Zhou G, Li F (2010). Anchoring hydrous RuO2 on graphene sheets for high‐performance electrochemical capacitors. Adv Funct Mater.

[7] Barakat NAM, Motlak M, Elzatahry AA, Khalil KA, Abdelghani EAM (2014). Ni_*x*_Co_1 − *x*_ alloy nanoparticle-doped carbon nanofibers as effective non-precious catalyst for ethanol oxidation. Int J Hydrog Energy.

[8] Yousef A, Akhtar MS, Barakat NAM, Motlak M, Yang OB, Kim HY (2013). Effective NiCu NPs-doped carbon nanofibers as counter electrodes for dye-sensitized solar cells. Electrochim Acta.

[9] Barakat NA, Motlak M (2014). Co_*x*_Ni_*y*_-decorated graphene as novel, stable and super effective non-precious electro-catalyst for methanol oxidation. Appl Catal B.

[10] Barakat NA, Motlak M, Lim BH, El-Newehy MH, Al-Deyab SS (2014). Effective and stable CoNi alloy-loaded graphene for ethanol oxidation in alkaline medium. J Electrochem Soc.

[11] Barakat NAM, Abdelkareem MA, Kim HY (2012). Ethanol electro-oxidation using cadmium-doped cobalt/carbon nanoparticles as novel non precious electrocatalyst. Appl Catal A.

[12] Barakat NAM, Shaheer Akhtar M, Yousef A, El-Newehy M, Kim HY (2012). Pd-Co-doped carbon nanofibers with photoactivity as effective counter electrodes for DSSCs. Chem Eng J.

[13] Zhang LL, Zhao X (2009). Carbon-based materials as supercapacitor electrodes. Chem Soc Rev.

[14] Zhang Y, Ren L, Wang S, Marathe A, Chaudhuri J, Li G (2011). Functionalization of graphene sheets through fullerene attachment. J Mater Chem.

[15] Sun Y, Wu Q, Shi G (2011). Graphene based new energy materials. Energy Environ Sci.

[16] Li L, Qiu J, Wang S (2013). Three-dimensional ordered nanostructures for supercapacitor electrode. Electrochim Acta.

[17] Ruiz V, Santamaría R, Granda M, Blanco C (2009). Long-term cycling of carbon-based supercapacitors in aqueous media. Electrochim Acta.

[18] Fuente E, Menendez J, Suarez D, Montes-Moran M (2003). Basic surface oxides on carbon materials: a global view. Langmuir.

[19] Jurewicz K, Vix-Guterl C, Frackowiak E, Saadallah S, Reda M, Parmentier J (2004). Capacitance properties of ordered porous carbon materials prepared by a templating procedure. J Phys Chem Solids.

[20] Frackowiak E (2007). Carbon materials for supercapacitor application. Phys Chem Chem Phys.

[21] Xu X, Zhou Y, Yuan T, Li Y (2013). Methanol electrocatalytic oxidation on Pt nanoparticles on nitrogen doped graphene prepared by the hydrothermal reaction of graphene oxide with urea. Electrochim Acta.

[22] Vinayan B, Sethupathi K, Ramaprabhu S (2013). Facile synthesis of triangular shaped palladium nanoparticles decorated nitrogen doped graphene and their catalytic study for renewable energy applications. Int J Hydrogen Energy.

[23] Xin Y (2012). Liu J-g, Jie X, Liu W, Liu F, Yin Y, et al. Preparation and electrochemical characterization of nitrogen doped graphene by microwave as supporting materials for fuel cell catalysts Electrochim Acta.

[24] Shao Y, Yin G, Gao Y, Shi P (2006). Durability study of Pt/C and Pt/CNTs catalysts under simulated PEM fuel cell conditions. J Electrochem Soc.

[25] Maldonado S, Stevenson KJ (2005). Influence of nitrogen doping on oxygen reduction electrocatalysis at carbon nanofiber electrodes. J Phys Chem B.

[26] Dommele S, áde Jong KP (2006). Nitrogen-containing carbon nanotubes as solid base catalysts. Chem Commun.

[27] Pietrzak R, Jurewicz K, Nowicki P, Babeł K, Wachowska H (2007). Microporous activated carbons from ammoxidised anthracite and their capacitance behaviours. Fuel.

[28] Wang H, Casalongue HS, Liang Y, Dai H (2010). Ni(OH)_2_ nanoplates grown on graphene as advanced electrochemical pseudocapacitor materials. J Am Chem Soc.

[29] Xing ZC, Chu QX, Ren XB, Tian JQ, Asiri AM, Alamry KA (2013). Biomolecule-assisted synthesis of nickel sulfides/reduced graphene oxide nanocomposites as electrode materials for supercapacitors. Electrochem Commun.

[30] Wang D, Kou R, Choi D, Yang Z, Nie Z, Li J (2010). Ternary self-assembly of ordered metal oxide-graphene nanocomposites for electrochemical energy storage. ACS Nano.

[31] Du X, Wang C, Chen M, Jiao Y, Wang J (2009). Electrochemical performances of nanoparticle Fe3O4/activated carbon supercapacitor using KOH electrolyte solution. J Phys Chem C.

[32] Zhang Y, Wang S, Li L, Zhang K, Qiu J, Davis M (2012). Tuning electrical conductivity and surface area of chemically-exfoliated graphene through nanocrystal functionalization. Mater Chem Phys.

[33] Marcano DC, Kosynkin DV, Berlin JM, Sinitskii A, Sun Z, Slesarev A (2010). Improved synthesis of graphene oxide. ACS Nano.

[34] Castrillón M, Mayoral A, Magén C, Meier J, Marquina C, Irusta S (2012). Synthesis and characterization of ultra-small magnetic FeNi/G and NiCo/G nanoparticles. Nanotechnology.

[35] Peng H, Mo Z, Liao S, Liang H, Yang L, Luo F (2013). High performance Fe- and N-doped carbon catalyst with graphene structure for oxygen reduction. Sci Rep.

[36] Li L, Zhang X, Qiu J, Weeks BL, Wang S (2013). Reduced graphene oxide-linked stacked polymer forests for high energy-density supercapacitor. Nano Energy.

[37] Barakat NAM, Khalil AK, El-Deen AG, Kim HY (2013). Development of Cd-doped Co nanoparticles encapsulated in graphite shell as novel electrode material for the capacitive deionization technology. Nano-Micro Lett.

[38] El-Deen AG, Barakat NA, Kim HY (2014). Graphene wrapped MnO_2_-nanostructures as effective and stable electrode materials for capacitive deionization desalination technology. Desalination.

[39] El-Deen AG, Barakat NA, Khalil KA, Kim HY (2013). Development of multi-channel carbon nanofibers as effective electrosorptive electrodes for a capacitive deionization process. J Mater Chem A.

[40] Sun L, Wang L, Tian C, Tan T, Xie Y, Shi K (2012). Nitrogen-doped graphene with high nitrogen level via a one-step hydrothermal reaction of graphene oxide with urea for superior capacitive energy storage. RSC Adv.

[41] El-Deen AG, Barakat NA, Khalil KA, Kim HY (2014). Hollow carbon nanofibers as an effective electrode for brackish water desalination using the capacitive deionization process. New J Chem.

